# Psychological Treatments for People With Severe and Enduring Anorexia Nervosa: A Mini Review

**DOI:** 10.3389/fpsyt.2020.00206

**Published:** 2020-03-24

**Authors:** James Zhu, Yive Yang, Stephen Touyz, Rebecca Park, Phillipa Hay

**Affiliations:** ^1^South West and North Coast Psychiatry Training Network, Sydney Local Health District, Sydney, NSW, Australia; ^2^School of Health Sciences, University of Newcastle, Callaghan, NSW, Australia; ^3^InsideOut Institute, Charles Perkins Centre, University of Sydney, Sydney, NSW, Australia; ^4^School of Psychology, University of Sydney, Sydney, NSW, Australia; ^5^Department of Psychiatry, Warneford Hospital, University of Oxford, Oxford, United Kingdom; ^6^Western Sydney University, Translational Health Research Institute, Sydney, NSW, Australia; ^7^South Western Sydney Local Health District, Camden and Campbelltown Hospitals, Sydney, NSW, Australia

**Keywords:** eating disorder anorexia nervosa, therapy, treatment resistant, longstanding chronic, therapy

## Abstract

This Mini-Review presents recent research into evidence for psychological treatments for people with severe and enduring anorexia nervosa (SEAN). Two psychological therapies, specialist supportive clinical management (SSCM), and cognitive behavior therapy for anorexia nervosa (CBT-AN) have limited (one randomized controlled study) evidence of efficacy. Both have had adaptations for SEAN, notably with revision of the primary treatment goal of improved quality of life and full weight recovery a secondary goal. A major issue with existing studies is poor definition of SEAN, and the large deficit in research that has used an agreed definition of SEAN. In particular, it may be problematic to extrapolate from studies of people with either severe and/or enduring but not intractable or “resistant” illness. People with longstanding AN who have not received evidence based care should be offered this with an expectation of recovery. Similarly, people with SEAN may be offered care with judicious mitigation of expectations. In the future, trials should include people with SEAN clearly defined. Trials with a subsample of participants likely to have SEAN, if identified at randomisation, are an opportunity for secondary analyses of such participants. This would widen the evidence base for psychological treatments providing hope for people with this devastating illness. Finally, there is an urgent need not only to strengthen our existing knowledge with studies of sufficient power, but also, fundamentally, to derive novel conceptualizations of what “treatment” involves.

## Introduction

Anorexia nervosa (AN) is a serious mental disorder affecting 1.4% of women and 0.2% of men worldwide in their lifetime (1). A significant number of people with AN remain ill for many years, with treatment becoming increasingly challenging and expensive as the disease progresses and recovery is less likely (2, 3). These individuals have the highest mortality rate of all mental disorders and suffer personal and fiscal impoverishment (4, 5). It is essential to stress that both treatment and recovery may occur decades after onset and that illness should not be regarded as intractable merely because of a lengthy duration (2, 6). Thus, it is important to approach the assessment of people with AN holistically.

In this paper the proposed criteria for severe and enduring AN (SEAN) [(7), p. 2] will be used. These are the presence of: “(1) a persistent state of dietary restriction, underweight, and overvaluation of weight/shape with functional impairment; 2) duration of > 3 years of anorexia nervosa; and, (3) exposure to at least two evidence based treatments appropriately delivered together with a diagnostic assessment and formulation that incorporates an assessment of the person's eating disorder health literacy and stage of change.” One major problem with these criteria is that the mean duration of illness in adults at the time of first presentation is frequently much more than 3 years, and some definitions of SEAN include an illness duration of > 7 years. Notwithstanding this limitation, these criteria capture the concept of SEAN as used in this paper.

Approaches to the care of people with SEAN have to date been based on consensus judgments and “clinical wisdom” with few clinical trials (8). There are mixed views on goals of treatment, with some suggesting these should be modified to aim for improved quality of life as a primary outcome (9, 10) whilst others promote a view that the aim of full weight recovery should remain the primary goal no matter the length or severity of illness (11). When undertaking treatment in those with SEAN, a unique challenge is posed for clinicians as patients are very unwell, often appear poorly motivated, feel very alone and unsupported, and experience an overwhelming sense of hopelessness (12). These people experience unremitting and insistent ruminations on food, shape, and weight and treatments aiming for “complete cure” may have little impact. Singularly offering treatments focused on traditional goals (primarily of weight restoration and amelioration of disordered eating symptoms) and physical and psychological recovery may be inappropriate for such individuals, and this mismatch in clinician and patient goals could further contribute to low retention rates (8).

A systematic review in 2012 (13) of randomized controlled trials (RCTs) highlighted the paucity of evidence based treatment for SEAN. This review included participants with AN and an illness duration of 3 or more years. It identified 11 trials, of which four had a majority of such participants. Findings were very limited and there was an absence of evidence for any psychological treatment to be first line in treatment, in contrast to medication which has generally been found to be non-efficacious or non-feasible as a first-line treatment. It was concluded that there may be an advantage for specialist *vs* non-specialist care and cognitive behavior therapy for AN (CBT-AN) may reduce relapse when used following weight restoration. All studies of any treatments required replication and there was no RCT specifically for treatment of people with SEAN.

The 2012 review (13) did, however, identify several psychological therapies that appeared to address issues relevant to people with SEAN, namely comorbidities of mood intolerance and depression, functional impairments, personality vulnerabilities and interpersonal deficits, low motivation to change, and/or modification of treatment goals towards quality of life. These therapies were: Specialist Supportive Clinical Management [SSCM (10)], CBT-AN (11), the Community Outreach Partnership Program [COPP (14)], Maudsley Model of Anorexia Nervosa Treatment for Adults [MANTRA (15)], and that described by Strober (12). A transdiagnostic CBT enhanced–broad [CBT–Eb (16)] therapy was also included as it widened the components of treatment to add modules addressing comorbidities, interpersonal therapy, clinical perfectionism, and low self-esteem. There are shifts towards more collaborative treatment and aims to improve quality of life, however, all these therapies emphasize the importance of ensuring medical safety and encourage, if not mandate, weight gain. Finally, only SSCM, CBT-AN, MANTRA, and CBT-E have been tested in a RCT against a control (active or inactive) treatment for people with AN.

The lack of evidence based treatments for SEAN may have negative effects and result in loss of hope for both patients and clinicians, leading them to seek alternative and less conventional treatment goals and therapies. Wonderlich et al. (8) stated that these attempts to accommodate treatment for SEAN patients may result in “relatively unfocused, intermittent, supportive interventions, where goals become unclear and monitoring of clinical status becomes impressionistic and imprecise.” There is little to guide clinicians in challenging areas such as when to use involuntary treatment and the ethics of, and role of, non-specific medical palliation in these patients (17, 18). Strober (2004) reasoned that the objective of care in patients with SEAN may be to support the patient and make up for the effects of the disease—a palliative, holding management that offers support and comfort to partially alleviate the effects of the disease (12).

Given the challenging nature of SEAN treatment, researchers and clinicians are now exploring novel biological treatments based on a neuroscientific understanding of the disorder (19). These include brain-directed treatments such as neuromodulation with non-invasive (20) and deep brain stimulation (21, 22). Novel medications such as dronabinol, a synthetic cannabinoid (23) have also begun to be investigated. However, even if such experimental adjunctive biological approaches are found to improve patient outcomes, they should always be considered adjunctive strategies, used in combination with evidence based psychological treatments, and in accordance with sound ethical guidelines (24).

Since the 2012 systematic review (13) there have been further RCTs of treatment for people with SEAN and a Cochrane review of psychological therapies is in progress (21). This Mini-Review aims to (1) provide an overview of psychological treatment trials and other studies identified since the 2012 review in the course of a recent literature search (25), and (2) discuss treatment gaps and future research directions. Therapy trials are considered that were either a primary RCT for people with SEAN or included a substantive proportion of people with long-standing illness (e.g., median illness of more than 3 years) who may represent a subgroup of people with SEAN. In this Mini-Review inpatient as well as outpatient trials are considered. An overview included trials is found on **Table 1**.

**Table 1:** Trials identified in this review of psychological therapies that included a substantive proportion of people with long-standing anorexia nervosa (AN).

Trial	Psychological Therapy/ies	Sample Size (*n*)	Duration of Illness	Randomization	Allocation Concealment	Notes
Touyz (9)*	Cognitive Behavior Therapy (CBT) for Anorexia Nervosa Specialist Supportive Clinical Management(SSCM)—both modified for severe and enduring	63	Mean 16.6 years (SD 8.5)	Adequate Used Ephron's biased coin approach stratified within sites based on subtype of illness (restricting vs binge-purge type) and use of psychiatric medication.	Adequate Central randomization performed by biostatistician independent from intervention sites.	Therapists conducted both forms of treatment to control for no-specific therapist effects. Outcome assessments conducted by assessors masked to treatment allocation.
Dalle Grave (26)	CBT-Enhanced focused, CBT-Enhanced broad	80	Median 5.0 years (Range 0–26)	Adequate Computer-based minimization algorithm used to allocate patients into two programs (balancing age, gender, diagnosis, and BMI). When groups evenly balanced, pre-prepared block randomization list of varying size used to allocate patients.	Adequate Author H.D. (who did the computer-based minimization algorithm) was independent from recruitment.	Therapists conducted both types of treatment. Assessors blind to group.
Schmidt (27)	Maudsley model of Anorexia Nervosa Treatment for Adults (MANTRA), SSCM	72	Mean 80.6 months (SD 71.8)**	Likely adequate but did not specify what type of randomization code was used. Randomized after baseline assessment. Randomization codes from a computerized system were used—stratified by eating disorder severity.	Adequate Researcher independent from trial generated randomization codes. Treatment assignment codes contained in numbered sealed opaque envelopes. Administrator was notified of allocation and letter sent to inform patient.	Outcome assessments conducted by two assessors masked to treatment allocation. Participants reminded not to reveal treatment allocation to assessor. Masking success was tested and deemed successful.
Zipfel (28)	Focal Psychodynamic Therapy for AN, CBT -E, optimized Treatment as Usual (TAU)	242	≤6 years: 148 patients >6 years: 94 patients	Adequate Independent coordination center for clinical trials performed centralized randomization. Used the Rosenburg and Lachin covariate-adaptive randomization procedure. Stratified by center and duration of illness.	Adequate After randomization into groups, independent center faxed trial sites the treatment allocation.	Complete masking not feasible as 1/3 of patients were allocated to TAU.
Dingemans (29)	CRT+TAU, TAU	82	≤7 years: 44 patients >7 years: 38 patients	Adequate Randomization sequence created using SPSS v19, stratified by center and treatment unit with 1:1 allocation using random block sizes of 4. Individual not involved performed randomization.	Adequate Individual not involved in recruitment performed randomization. After assessment, neutral person not involved informed patients their assignment.	Researchers who were not involved in conducting CRT conducted assessments and were blind for condition.
Brockmeyer (30)	Cognitive Remediation Therapy (CRT), Non-specific Neurocognitive Therapy (NNT)	40	CRT (n = 20): *Mean 7.89 years (SD 7.60) NNT (n = 20): *Mean 6.82 years (SD 7.57)	Adequate Treatment assignment determined by independent research coordinator using specific open-source randomization software (RANDI2) stratified by duration of illness.	Adequate	Blinding of patients and therapists not possible.
Steinglass (31)	AN-Exposure and response prevention (EXRP), CRT	32	Mean 10 years (SD 8)***	Adequate Used computer generated block randomization procedure.	Unclear Patients informed of assignment after baseline assessments.	All study therapists provided both treatments.
Williams (14)	Community Outreach Partnership Program (COPP)	31	Mean 15.23 years (SD 8.15)	Not applicable	Not applicable	NA
Steinglass (32)	Supportive psychotherapy (SPT), Regulating Emotions and Changing Habits (REaCH)	22	SPT (n = 11): *Mean 12.7 years (SD 10.1) REaCH (n = 11): *Mean 15.5 years (SD 11.4)	Adequate Random assignment made using computer generated block randomization procedure.	Unclear Patients informed of treatment procedure after baseline assessments.	Assessors blind to treatment group.
Weiss (33)	Motivational Interviewing (MI), TAU	32	Mean 10.7 years (SD 8.9)	Adequate Randomized through permuted block randomization.	Unclear	Treatment staff in MI program kept blind to patient group assignment. Outcome assessment not blind to group.

*Trial designed to assess psychological therapy in people with SEAN.

**Data from 58 of 72 patients.

***Data from 30 of 32 patients.

## Psychological Treatment Trials

### Primary Trial of SEAN

To our knowledge there has been only one RCT evaluating specific psychological treatment for people with SEAN (9, 10). This trial compared two evidence based treatments for AN that were modified for people with SEAN, namely SSCM (34) and CBT-AN (35). In this study SEAN was defined as having a minimum illness duration of 7 years. In both treatment conditions there was a broadening of treatment goals to focus on quality of life and a lessening of the priority to achieve weight gain. Both therapies also highlighted the importance of collaborative goal setting to address patient preferences, with the aim to improve engagement, motivation, and retention rates. This trial did achieve very high rates of treatment completion—with 76% completing more than 30 weeks of therapy. There were significant improvements in eating disorder symptoms, health related quality of life and weight for all participants, which were sustained to 1-year follow-up. Whilst there were few significant differences between groups in primary or secondary outcomes, those with more severe symptoms, depression, an older age, and who were purging, benefited more from the modified CBT-AN (36). CBT-AN was also superior to SSCM in reducing core eating disorder symptoms at follow-up. For all participants improved eating disorder symptoms and increased weight also predicted a significantly better health related quality of life (10).

### Trials That Included Participants With Long-Standing Illness

#### Dalle-Grave CBT-E Trial

In 2013, Dalle Grave et al. (26) reported the immediate and longer-term effects of CBT-E in focused and broad forms in 80 young adults with severe AN and a median illness duration of 5 years. In this RCT both treatments significantly improved weight, eating disorder and general psychopathology in participants, and whilst deterioration did occur after discharge, it was not marked and only for a short duration. Furthermore, as there were no statistically significant differences between the two therapies, it was suggested that there appears to be no benefit of using the more complex CBT-Eb treatment. Thus, whilst Hay et al. (13) postulated that the broader form of CBT-E would offer benefits over the focused form for people with longer illness duration, this was not supported in this study. This may have been because SEAN encompasses the concept of previous treatment intractability as described above, and not merely longstanding symptoms. This trial was also conducted in an inpatient treatment setting where there was additional nutritional support from dietitians. Interestingly no psychotropic drugs were prescribed.

#### Maudsley Model of Anorexia Nervosa Treatment for Adults (MANTRA) Trials

MANTRA has not been evaluated in a SEAN group. However, it employs a motivational interviewing style, has as a model individualized care, and optional modules can include the development of a “non-anorexic” identity that was conceived for patients with enduring illness. (27, 37). MANTRA has been evaluated in two RCTs against SSCM in participants with lengthy (mean 8 and 7 years) illness durations. In these RCTs both therapies resulted in statistically significant improvements in BMI, reductions in ED symptomatology, other psychopathology, and clinical impairment over time. Notably, at 2-year follow-up (37) MANTRA was assessed to be statistically significantly more acceptable and credible by patients at 12 months, with higher treatment completion rates. This is a relevant finding as patients with SEAN have notoriously high attrition rates.

#### The Anorexia Nervosa Treatment of OutPatients (ANTOP) Trial

A treatment more recently evaluated in RCTs in AN is focal psychodynamic therapy (FPT). FPT is a three-phase treatment that focuses on interpersonal relationships (28). In FPT initially a therapeutic relationship is developed and self-esteem, pro-anoretic behavior, and ego-syntonic beliefs are addressed. Treatment then shifts to target associations between AN behaviors and interpersonal relationships. Finally preparations are made for independent recovery. The Anorexia Nervosa Treatment of OutPatients [ANTOP (28)] study was a large (n = 242) multi center RCT aimed to assess the efficacy of FPT and CBT-E against an optimized treatment as usual (TAU). Ninety-four (39%) of participants had AN for longer than 6 years. At the end of treatment (10 months after start of treatment) all groups had gained weight with no difference in weight gain between groups. All participants also exhibited decreased general and eating disorder-specific psychopathology. At 12-month follow-up, people allocated to FPT had significantly higher full recovery rates compared with those assigned to optimized TAU as usual, but there was no significant difference in recovery rates between the CBT-E and FPT groups. Moreover, CBT-E was found to be more effective in terms of speed of weight gain and improvements in eating disorder psychopathology and appeared to offer an advantage for those with lower baseline weight.

#### Cognitive Remediation Therapy (CRT)

CRT was initially a form of management for patients with brain lesions and schizophrenia. A modified form of CRT was developed by Tchanturia et al. (38) for AN. It is a focused treatment that targets a patient's cognitive inflexibility, thought to be a factor in both the development and maintenance of the illness. With inefficient cognitive flexibility, patients may exhibit obsessive preoccupations related to body shape, weight, and food as well as ritualistic behaviors. Through improvement of basic neurocognitive functions, it is thought that perseverative behaviors in anorexia nervosa may be addressed. Instead of looking at what a patient thinks, *how* they think is examined and it is thought that the proliferation of neural connections due to CRT training results in more flexible thought and behaviors. In an RCT, Dingemans et al. (29) investigated the effectiveness of 10-session CRT for 82 patients with severe or enduring eating disorders by comparing intensive TAU to CRT plus TAU. Forty-six percent of participants had >7 years of illness duration and 93% had a history of AN. Patients who underwent CRT in addition to TAU improved significantly more in eating disorder-related quality of life at end of treatment and eating disorder psychopathology at follow-up when compared to TAU only patients. Whilst Dingemans et al. (29) confirmed the findings of previous uncontrolled case studies using CRT, it should be noted that this trial was not in SEAN patients only, but rather a mix of severe or enduring eating disorder patients. No studies have been conducted with CRT in exclusively SEAN patients.

Brockmeyer et al. (30) also have investigated CRT in a small pilot RCT of people with AN with mean illness duration > 6 years. They only examined outcomes in the 25 participants who completed treatment and only reported on neurocognitive findings. CRT was compared with a non-specific neuro cognitive therapy that focused solely on attention, memory, and deductive reasoning, and not flexibility. The primary outcome was cognitive set-switching which was superior at end of treatment in the CRT group.

#### Exposure and Response Prevention for Anorexia Nervosa

Exposure and Response Prevention for AN (AN-EXRP) is a manualized method of treatment that was formed to focus on eating-related anxieties and alter dysfunctional eating behaviors through encouraging the confrontation of fears. (31) AN-EXRP modifies these strategies to target eating-related symptoms with particular focus on exposure and ritual prevention. Patients are progressed through a hierarchy of provocative eating situations and attention is brought to stopping ritualistic behaviors and the eventual dissipation of anxiety. Steinglass et al. (31) performed an RCT to evaluate AN-EXRP as an adjunctive strategy to improve eating behavior during weight restoration and compared it with CRT. This study was conducted with participants with AN and a mean illness duration of 10 years. Participants randomized to AN-EXRP had significantly better change in food intake in a test meal than CRT (i.e. average intake increased by approximately 50 kcal compared to a decrease of 77 kcal in the CRT group). Whilst these findings could suggest that AN-EXRP may support continuation of healthy behaviors, a key limitation of the study was that the intervention was delivered adjunct to an intensive treatment and so results cannot be attributed to AN-EXRP alone.

#### Community Outreach Partnership Program (COPP)

COPP was developed as an outpatient, multidisciplinary form of treatment that differed to traditional approaches in that its goals were improving quality of life instead of targeting the eating disorder. (14) Patient autonomy and self-esteem are improved through skill building and problem solving, and support systems that favor community supports instead of traditional health care providers and hospitals, are developed. A harm reduction model is utilized, where there is collaboration between patient and therapist in an attempt to minimize consequences that may arise from disordered eating behaviors. The program employs strategies from psychosocial rehabilitation and motivational interviewing. Preliminary outcome results have been reported from a small uncontrolled study by Williams et al. (14) in patients with eating disorders (mean duration of eating disorder 15.23 years), 15 (48%) of which were patients with AN. Positive findings suggested that COPP merits further evaluation in RCTs as an alternative model of care for individuals with long-standing eating disorders.

#### Regulating Emotions and Changing Habits (REaCH)

REaCH is a behavioral treatment focused on prompts for disordered eating behaviors and was based upon treatments that have seen success in complex behaviors such as habit reversal therapy for Tourette's syndrome. (32) It has been postulated that patients with AN develop restrictive eating behaviors that eventually grow into entrenched reflex responses to certain cues and thus are considered habits. REaCH is a manualized treatment with four main components including cue-awareness, the development of new routines, the repression of detrimental habits, and emotional regulation. In a proof-of-concept RCT by Steinglass et al. (32) 22 hospitalized patients with AN were randomly assigned to either Supportive Psychotherapy (SPT) or REaCH (mean duration of illness was 12.7 and 15.5 years respectively). Study findings demonstrated that REaCH more effectively altered habit strength of maladaptive routines compared to SPT and was associated with clinically meaningful improvements in eating disorder symptoms and energy intake.

#### Motivational Interviewing (MI)

Weiss et al. (33) tested a four-session weekly MI therapy prior to an intensive hospital program in 39 eating disorder participants (65% with AN) with mean illness duration >10 years. The primary outcome was program completion which was significantly higher in the MI group than a control (wait list) arm. However, there were no differences in measures of motivation and thus the mechanism of the effect was unclear.

## Discussion

Whilst there has been some progress in psychological treatment research in AN there remains a grave paucity of trials, and a total of one only (9) for those with severe enduring illness. The situation is further confused by lack of staging patient's illness. As many trials include participants who may have SEAN there is an opportunity to stratify by presence or absence of SEAN and conduct *post-hoc* sub-group analyses in large trials. However, this rests on an agreed definition of SEAN and it is likely the third suggested criterion of treatment intractability would not be known and/or would have been met, which limits this as a means of advancing knowledge. Indeed, it was not a criterion in the single trial of SEAN (9). There is an urgent need not only to strengthen our existing knowledge through larger RCTs of sufficient power, but also, fundamentally, to derive novel conceptualizations of what “treatment” involves, beyond traditional directive and paternalistic models of the past which all too often render patients institutionalized, traumatized, and/or disempowered.

There needs to be an increased effort in determining how this particular population of patients is best empowered to recover. This should be mindful of respecting patients' preferences which are too often neglected in this area (39). Unanswered research questions include: what are the moderators of specific therapies so we can better manage patients with different presentations; how can we avoid providing ineffective treatment, and thus periods of undernutrition, to patients who are unlikely to respond to a certain treatment; what is the role of new technologies such as transcranial direct current stimulation in delivering psychotherapy and support to these chronic patients; and, might there be a more suitable and accurate way to categorize anorexia nervosa such as the hybrid model of categories and dimensions suggested by Wildes (40)?

In conclusion, advances have been made in bringing to public awareness the distinction between acute presentations of AN and that of the severe and less tractable kind. There are several psychological therapies with an emerging evidence base that can be further tested and adapted for SEAN treatment. Research to date, albeit minimal, provides hope for the emergence of new concepts and a stronger evidence base to guide treatments.

## Author Contributions

PH, ST, and RP conceived this paper. JZ and PH reviewed the literature. JZ and YY wrote the first draft of the manuscript. JZ, YY, ST, RP, and PH edited and provided intellectual input to the manuscript.

## Conflict of Interest

PH receives/has received sessional fees and lecture fees from the Australian Medical Council, Therapeutic Guidelines publication, and New South Wales Institute of Psychiatry and royalties/honoraria from Hogrefe and Huber, McGraw Hill Education, and Blackwell Scientific Publications, Biomed Central and Plos Medicine and she has received research grants from the NHMRC and ARC. She is Chair of the National Eating Disorders Collaboration Steering Committee in Australia (2019–) and was Member of the ICD-11 Working Group for Eating Disorders (2012–2018) and was Chair Clinical Practice Guidelines Project Working Group (Eating Disorders) of RANZCP (2012–2015). She has prepared a report under contract for Shire Pharmaceuticals in regard to Binge Eating Disorder (BED; July 2017) and Honoraria for training Psychiatrist in BED assessment. ST receives royalties for written work from Hogrefe and Huber, Taylor and Francis and Routledge. He is the Chair of the Shire/Takeda Clinical Advisory Committee for Binge Eating Disorder, has receive honoraria from Shire/Takeda for commissioned reports, as well as both travel and research grants. He is a consultant to Weight Watchers (WW). ST is a mental health adviser to the Commonwealth (of Australia) Department of Veteran's Affairs. ST is a member of the Commonwealth of Australia's Department of Health Technical Committee for Eating Disorders. He is Editor of the Journal of Eating Disorders.

The remaining authors declare that the research was conducted in the absence of any commercial or financial relationships that could be construed as a potential conflict of interest.
